# Supplementary material for: Spatial-temporal characterization of cropland abandonment and its driving mechanisms in the Karst Plateau in Eastern Yunnan, China, 2001–2020
Source: PLoS One. 2024 Jul 17;19(7):e0307148. doi: 10.1371/journal.pone.0307148 (PMC11253946; doi:10.1371/journal.pone.0307148)
Supplement: Attachment — Submitted filename: Response to Reviewers.docx [file pone.0307148.s001.docx]

Dear Editor:

We really appreciate all of the insightful and constructive comments from the reviewers. We have thoroughly revised the manuscript (PONE-D-23-43715R1) according to all comments received. In our point-by-point response attached below, reviewers’ comments is in larger italic fonts (12 pt.) and our response is in smaller fonts (10 pt.). We hope that the revised version would be closer for publication in your journal. Look forward to hearing from you. Thank you.

With best wishes,

Yours sincerely,

Jiasheng Wang

Response to reviewers’ comments on our manuscript

PONE-D-23-43715R1

**Reviewer #1**

1. *Identifying agricultural lands in the Yunnan Plateau.*

**Reply:**

We've added information about agricultural lands in the Yunnan Plateau in Line 134 of the modified manuscript. The added content is as follows.

**Added content**:

The cropland area in KPEYC accounts for 35% of the province's cropland.

2. *Adding recent scientific papers on the abandonment of agricultural lands in China and in that region by combining temporal and spatial studies on the abandonment of agricultural lands.*

**Reply:**

Thanks for your suggestion. We added references in line 102. The supplementary references are as follows.

**Added content**:

Line 102,

From the perspective of research methods, the existing research on cropland abandonment lacks an analysis of the driving mechanism of spatial and temporal changes. From the perspective of driving factors, existing studies mainly analyze the driving mechanisms of cropland abandonment in terms of parcels or households**^[19,20,21]^**.

[19] HAN Dejun., YAN Zhao., SONG Yang. Study on the two-way effect of "alleviating abandonment" and "intensifying non-food" of agricultural land system innovation--The example of "three changes" reform in southwest mountainous area[J]. China Land Science,2023,37(09):73-84.

[20] QUAN Zhijun, WEN Jun, LI Pengju. Research on the status quo and countermeasures of arable land protection in the context of food security--Taking Yibin City as an example[J]. Southern Agricultural Machinery,2023,54(24):175-177.

[21] YANG Ruixue,PENG Haiying. A review of domestic and international research on cropland abandonment[J]. Journal of Management Cadre College of Ministry of Agriculture and Rural Affairs,2023,14(03):59-65.

*3. Line 46: The topic is vital and a recommendation must be added to the research based on the results obtained*

**Reply:**

Thank you for your review and comments. In this paper, we have changed it according to your suggestion in line 45-51.

**modified content:**

Based on the results, this study can provide decision-making support for local prevention and control of cropland abandonment, and the local community needs to promote land transfer and concentration and local urbanization according to local conditions, improve agricultural policies, improve farming conditions, etc. in order to increase farmers' enthusiasm for production, promote the rational use of cropland, and solidly push forward ecological restoration and management, optimize ecological spatial patterns, manage serious areas of rocky desertification, and appropriately alleviate the contradiction between people and land.

4. *Line 66: reference* *#9 missed.*

**Reply:**

Thank you for your review and comments. References *#9* have been added in line 71 of the modified manuscript.

**modified content:**

Scholars have conducted extensive research on the driving mechanisms and influencing factors, mainly analyzing the factors of abandonment from the perspective of the social system and land use change**^[8,9,10,11]^**.

5. *Line 147: add i at the end “the year before the year i”;*

**Reply:**

Thanks for your valuable counsel. We have added the add i at the end. The modified content is as follows:

**modified content:**

where$AR_{i}$ is the CAR in the year$i$, $AA_{i}$is the area of cropland abandonment in the year$i$, and $CA_{(i-1)}$is the area of cropland in the year before the year$i$.

6. *Line 176: in the methods, the author did not explain the test used in testing the time series.*

**Reply:**

Thank you again for reviewing the manuscript so carefully. In response to the reviewer's questions regarding the time series testing method, we have now detailed the ADF test used in the methods section, including the purpose of the test, steps, and parameter settings. We believe these supplements will help readers better understand our research methods and results.

**added content:**

**3.2 Time series testing**

To test the stationarity of the abandoned cultivated land over time series, we employ the ADF (Augmented Dickey-Fuller test), a rigorous statistical testing method. It determines whether a time series is stationary by comparing the test statistic with its corresponding critical values. If the test statistic is less than the critical value and the p-value is less than 0.05, we can reject the null hypothesis and conclude that the time series is stationary. Results are shown in Table 2. As can be seen from Table 2, when the difference is at order 0, the significance P-value is 0.001, which indicates significance at the given level. Thus, the null hypothesis is rejected, and the series is considered to be a stationary time series.

**Table 2. ADF Time Series Test Results Table**

| variable | Difference Order | | t | P | AIC | 1% | 5% | 10% |
| --- | --- | --- | --- | --- | --- | --- | --- | --- |
| CAR | | 0 | -4.132 | 0.001 | -56.957 | -3.833 | -3.031 | -2.656 |
|  |  | 1 | -5.414 | 0.000 | -51.999 | -3.889 | -3.054 | -2.667 |
|  |  | 2 | -0.904 | 0.787 | -50.326 | -4.332 | -3.233 | -2.749 |

7. *Line 188: in the Economic Environment variables, the problem of linear correlation usually appears in such variables. The author did not indicate here the reason for adding these variables, knowing the existence of this problem.*

**Reply:**

Thank you for your comments the manuscript so carefully. The reason for selecting these variables is that these variables are directly related to cropland abandonment, and their relationship with cropland abandonment has been clearly explained in the Description column of Table 1. In the revised version, a table description has been added at the end of the last first paragraph of section 3.1. There is indeed a problem of linear correlation between economic environment factors. GDP is the sum of output value of the three industries, so the GDP factor has been removed from the revised draft, and the result has also been modified accordingly. There was no obvious linear correlation between the other variables.

8. *Line 188: There are about 18 previous studies that were mentioned on pages 3, 4, and 5, and the researcher must link them to the description*.

**Reply:**

Thank you for your comments the manuscript so carefully. In this paper, we mentioned here is that have referred to many references earlier, so in the Description section of Table 1, We linked to these references.

**modified content:**

| **Level**  **Indicators** | **Level 2**  **Indicators** | **Unit** | **Description** |
| --- | --- | --- | --- |
| **Population** | Year-end total population X1 | *Million people*  *Million people*  *Million people*  *Million people* | The larger the population, the less likely it is that cropland will be left fallow. **^[1]^** |
|  | Number of students in primary and secondary schools X2 |  | It can be used as supplementary labor for farming. **^[14]^** |
|  | Rural workers X3 |  | The lower the amount of labor, the higher the likelihood of abandonment. **^[5]^** |
|  | Agricultural population X4 |  | A decrease in the rural agricultural population makes abandonment more likely. **^[1]^** |
| **Economics**  **Environment** | Gross value of agricultural production X5 | *Billion yuan*  *Billion yuan*  *Billion yuan*  *Ten thousand yuan* | The greater the value of agricultural production, the more likely it is to be abandoned. **^[4]^** |
|  | Gross value of industrial production X6 |  | The more developed the industry is, the more likely it is to be abandoned. **^[7]^** |
|  | Gross tertiary industrial production X7 |  | The larger the output value of tertiary industry is, the more likely it is to be abandoned. **^[13]^**. |
|  | Per capita net income of farmers X8 |  | The higher the income of farmers, the higher the likelihood of abandonment. **^[5]^** |
| **Cropland**  **Attributes** | Soil thickness X9 | *Meter* | The thicker the soil layer, the less likely it is to be abandoned |
|  | Soil organic matter content X10 | *Grams per kilogram* | The higher the quality of the soil, the less likely it is to be abandoned. **^[5]^** |
|  | Average sunshine duration X11 | *Hour* | The better the light, the less likely it is to be abandoned. |
|  | Average air temperature X12 | *Celsius* | The better the temperature, the less likely to be abandoned. |
|  | Average precipitation X13 | *Millimeter* | The richer the water resources, the less likely it is to be abandoned. |
| **Farming**  **Conditions** | Elevation X14 | *Kilometer* | The higher the altitude, the more prone to abandonment. **^[14]^** |
|  | Slope X15 | *Degree* | The steeper the slope, the more likely it is to be abandoned. **^[11]^** |
|  | Slope direction X16 | *Degrees* | The sunnier the slope, the better the light, the less likely it is to be abandoned. **^[14]^** |
|  | Traffic mileage X17 | *Thousand Kilometers* | Poor access to cropland can lead to abandonment. **^[11]^** |
|  | Agriculture machinery powerX18 | *Million Watts* | The lower the degree of mechanization, the higher the likelihood of abandonment. **^[5]^** |

9. *Line 249: What does “ACR” mean?*

**Reply:**

Thank you for your detailed comments. I am sorry, this word is my spelling error, the correct word is CAR.

10. *Line 265: How were these proportions divided? Or what method is used in division?*

**Reply:**

Thank you so much for your careful reading. The CAR were divided into four classified: class I (CAR ranging from 0% to 4.445%), class II (CAR ranging from 4.445% to 7.85%), class III (CAR ranging from 7.85% to 12%), and class IV (CAR ranging from 12% to 70%). These proportions are divided according to the following content.

**added content:**

According to the quantity of the 20-year CAR of 28 counties in KPEYC, the abandonment degree of each county in each year is expressed by dividing it into 4 levels according to the equal interval method.

11. *Line 287: why the # of drivers decrease from 19 to 11 (author didn’t mention the reason).*

**Reply：**

Thank you so much for your careful reading. The reason for the reduction in the number of driving factors from 19 to 11 is mainly due to the fact that some attributes of the driving factors in the natural environment remain constant over a long period. If correlation analysis is performed, it cannot reflect their correlation with the rate of land abandonment. Therefore, the driving factors selected for Spearman correlation analysis are primarily those that vary over time.

**modified content:**

Due to the fact that certain attributes of driving factors in the natural environment remain constant over a long period, if correlation analysis is conducted, it cannot reflect their correlation with the CAR. Therefore, the driving factors selected for Spearman correlation analysis are primarily those that vary over time. In this paper, we take the entire study area as the unit of calculation and use correlation analysis to explore the degree of correlation of population, economic environment, two farming conditions factors and changes in CAR.

12. *Line 290-292: Do not forget correlation sign..*

**Reply:**

Thank you so much for your careful reading. We'll modify it with the suggestions you gave us.

**modified content:**

The results are shown in Figure 4. The variables examined are gross value of agricultural production (X5), gross value of industrial production (X6), gross tertiary industrial production (X7), and agriculture machinery power (X18). The correlation between economic factors and cropland abandonment is the largest, with $R_{X5}$ is 0.329, $R_{X6}$is 0.302, and$R_{X7}$ is 0.274. Agriculture machinery power, among the farming conditions, has a correlation value of 0.289 with the change in the CAR.

13. *Line 337: the result of X4 and X19 not equal to the numbers in figure 5*.

**Reply:**

Thanks for your suggestion. Due to my carelessness, there are errors in the data in Figure 5. The figure is modified as follows.

**modified content:**

14. *Line 355: there were some variables had high-value interaction detection not mention, such as X4 with X8 and X14 with X18.*

**Reply:**

Thanks for your suggestion. Due to my carelessness that I did not analyze these two pairs of interaction detection results. The relevant modifications have been highlighted in Figure 5, and since there are many combinators with large values of interaction detection q, the analysis in the text focuses on combinators with interaction factor values greater than 0.9.

15. *what does“o”; mean?*

**Reply:**

Thanks for your suggestion. I typed an extra "o" because of my carelessness.

**modified content:**

From the model, we can see that gross value of agricultural production, and gross tertiary industrial production among the economic environment factors have a significant effect on CAR.

16. *change the word “thesis”; to “study”.*

**Reply:**

Thanks for your suggestion. I change the word thesis to study.

**modified content:**

To analyze the spatial-temporal patterns in KPEYC, this study used correlation analysis and geodetector to identify the main factors that have driven the cropland abandonment in this region over time and space. By applying linear regression analysis to explore the driving mechanisms behind the spatial-temporal changes in cropland abandonment. The following conclusions were reached:

17. *Line 410: 9.78%.*

**Reply:**

Thanks for your suggestion. Taking into account your comments and those of the other reviewers, we have revised this section，and deleted 9.78%.

18. *Line 438: what does “KPTYC” mean?*

**Reply:**

Thanks for your suggestion. I change the KPTYC to KPEYC.

**modified content:**

To analyze the spatial-temporal patterns in KPEYC, this study used correlation analysis and geodetector to identify the main factors that have driven the cropland abandonment in this region over time and space. By applying linear regression analysis to explore the driving mechanisms behind the spatial-temporal changes in cropland abandonment. The following conclusions were reached:

19. *Cereal were not discussed in the research, so why did the result appear here? Note that the Cereal area is large.*

**Reply:**

Thanks for your question. Cereal is grain production in the research, and the last section is the policy recommendations made, fully incorporating your suggestions, and we have placed the policy recommendations section in the discussion section.

**modified content:**

According to the results of this study, it can still help the relevant departments to understand the status quo of regional cropland abandonment and provide corresponding data support for future cropland protection policies. It is necessary to create a different kind of countryside for “agriculture+ tourism”, promote land transfer and concentration according to local conditions, local urbanization, improve agricultural policies, and improve farming conditions in order to increase farmers' enthusiasm for production and promote the rational use of cropland; it is necessary to solidly push forward ecological restoration and governance, optimize the ecological spatial pattern, manage the serious areas of rocky desertification, and appropriately alleviate the contradiction between people and land. To establish a long-term mechanism to rectify the abandonment of cropland; to orderly promote the transfer of land in accordance with the law, to guide farmers who are not in a position to carry out normal farming to transfer land to the main body of the operation, and to encourage conditional places to carry out the market operation of land transfer, to revitalize the cropland resources, and to avoid the waste of land resources; at present, the governments of various levels of the abandonment of cropland have had a preliminary understanding of the rectification of cropland abandonment has had initial results, but the protection of cropland resources is a long-term process, so as to promote the rational use of cropland. The protection of cropland resources is a long-term process, to fully mobilize the enthusiasm of farmers to grow food, it is necessary to establish a long-term early warning of cropland abandonment, remediation mechanism, to put an end to the phenomenon of cropland abandonment of the resurgence of the phenomenon.

20. *Line 513: reference #9 not mention in the study.*

**Reply:**

Thank you for your review and comments. References *#9* have been added in line 71 of the modified manuscript.

**modified content:**

Scholars at home and abroad have conducted extensive research on the driving mechanisms and influencing factors, mainly analyzing the factors of abandonment from the perspective of the social system and land use change**^[8,9,10,11]^**.

**Reviewer #2**

1. *Lack of clarity in research objectives: The article does not clearly state the specific research objectives or hypotheses that the study aims to address.*

**Reply:**

Thanks for your constructive questions. We have added content on this issue.

**modified content:**

In summary, this study introduced the research progress, influencing factors and methods of cropland abandonment, but karst landscapes cover about 20% of the land surface, but there are very few studies on cropland abandonment in this particular landscape. The Karst Plateau in Eastern Yunnan, China (KPEYC) is one of the largest areas in China known for its karst landscape distribution. Cropland in the region is fragmented, the ecological environment is fragile, per capita cropland is small, the quality of cropland is generally not high, and there are insufficient reserve resources of cropland, so it is difficult to rely solely on agricultural incomes to support household expenditures，and pronounced human-land conflicts. As a result, many rural workers migrate to cities and towns for work, resulting in significant cropland abandonment. This study aims to address three aspects: (i) the spatiotemporal distribution characteristics of abandoned cropland; (ii) the main driving factors of cropland abandonment; (iii) the driving mechanisms of cropland abandonment in the eastern part of Yunnan Province.

2. *Insufficient methodological description: The article briefly mentions the use of correlation analysis, geodetector, and regression analysis methods, but it lacks a detailed description of how these methods were applied.*

**Reply**：

Thank you again for reviewing the manuscript so carefully. We have added content on this issue. For the correlation analysis we used the Spearman correlation analysis, whose method is described in the manuscript; the method of geodetection valence is also explained in more detail in the text; the regression analysis method was chosen to be stepwise regression, whose method is able to solve the linear correlation problem in the driving factors.

**added content:**

**3.2 Drivers of temporal changes of CAR**

They have not changed much over the years, as factors such as cropland attributes and farming conditions have remained relatively stable. In this paper, we take the entire study area as the unit of calculation and use correlation analysis to explore the degree of correlation of population, economic environment, two farming conditions factors and changes in CAR. It mainly involves the calculation of indicators and correlation coefficients.

(1) Indicator calculation. The average value of each indicator in each year is taken as the indicator value of the study area, and the time series of the indicator value is the independent variable *X*. The average value of the annual cropland abandonment area/abandonment rate in the region is taken as the dependent variable *Y*.

(2) Spearman correlation analysis **^[23]^**. Correlation analysis aims to determine the correlation coefficients between each variable and the dependent variable and to determine whether they are significant or not using statistical tests. Spearman's correlation coefficient is also known as the rank correlation coefficient, which requires two conditions to be met, 1) the variables contain ranked variables or the variables do not follow a normal distribution, 2) there is a monotonic relationship between the two variables. This approach has a wider range of applications than Pearson's correlation coefficient. The formula is as follows:

$R_{s}=\frac{\sum_{i=1}^{N} (P_{i}-\overline{P})(S_{i}-\overline{S})}{[\sum_{i=1}^{N} (P_{i}-\overline{P})^{2}\sum_{i=1}^{N} (S_{i}-\overline{S})^{2}]^{\frac{1}{2}}}=1-\frac{6\sum_{i=1}^{N} d_{i}^{2}}{N(N^{2}-1)}$ (2)

In the formula, $R_{s}$represents the correlation coefficient between the drivers and CAR, and the value range $R_{s}$ is [-1,1]. When$R_{s}>0$, the impact factor is positively correlated with the CAR; When$R_{s}<0$, the impact factor is negatively correlated with the rate of cropland abandonment. The larger the absolute value $R_{s}$, the greater the correlation between the impact factor and the CAR. $n$is the capacity of the sample; $P_{i}$ and $S_{i}$ represent the average values of the observed data$i$; $\overline{P}$ and$\overline{S}$ respectively represent the average ranks of the variables$x_{i}$ and$y_{i}$; and $d_{i}=P_{i}-S_{i}$.

**3.3 Drivers of spatial variation of CAR**

To explore the driving factors of the spatial variation of cropland abandonment, this paper analyzed the driving factors of the spatial distribution of cropland abandonment using the geodetector model**^[18]^** . Cropland abandonment is a complex process, and most of the existing studies of abandonment drivers have used traditional econometric models, focusing on the effects of individual factors on abandonment and paying little attention to the interactions of multiple factors. Geodetector are a set of statistical methods for detecting spatial heterogeneity and revealing the driving forces behind it. Each time-varying factor was calculated as the average value of 2001-2020 by county as the factor value. The invariant factor was added as the independent variable *X*, and the average value of the CAR from 2001 to 2020 was taken as the dependent variable *Y*. Geodetector was used to detect the driving factors of the spatial distribution of the average CAR.

(1) Spatial stratification and heterogeneity detection

The geodetector can detect the spatial stratified heterogeneity of the variable *Y* and the degree of explanation of a certain factor (*X*) on the spatial variability of the CAR (*Y*), which is measured by the q-value, and its calculation formula is equation (3):

$q=1-\frac{1}{n\sigma^{2}}\sum_{h=1}^{L} n_{h}\sigma_{h}^{2}$ (3)

Where L is the number of partitions of the variable$X$, $n$ is the sample size of $Y$, $\sigma^{2}$is $Y$the overall variance of $Y$,$n_{h}$ is the $h$sample size of the stratified samples$h$, and $\sigma^{2}$is the variance in the$h$ partitions. The$q$ of factors value range is [0,1], the larger the value, the stronger the explanatory power of the independent variable on the abandonment rate$Y$.

(2) Interaction detection

The geodetector also has the function of interaction detection, which can analyze the interaction between different factors, i.e., to assess whether the factors $X_{1}$ and$X_{2}$ together increase or decrease the explanatory power of the variable $Y$, and also to calculate whether the factors are independent of each other. The calculation method is to calculate the explanatory power of the two factors$q_{1}$ and$q_{2}$, and then calculate the $q$value of the new polygon formed by the tangent of the superposition of the polygon of the two variables, and compare it with the explanatory power of a single factor, and the relationship between the two factors is shown in Table 2.Interaction detectors detect interactions between factors, determine whether variable interactions exist between indicators, and determine the strength, direction, nonlinearity, and nonlinearity of interactions. In this study, an interaction detector was used to detect the effect of a superposition of cropland abandonment indicators on the CAR.

**Table 2.** Interactive detection between different factors

| Basis of judgment | Type of interaction |
| --- | --- |
| *q*(X1∩X2) < Min[*q*(X1), *q*(X2)] | Nonlinear strengthening and weakening |
| Min[*q*(X1), *q*(X2)] < *q*(X1∩X2) < Max[*q*(X1), *q*(X2)] | Single-factor nonlinear attenuation |
| *q*(X1∩X2) > Max[*q*(X1), *q*(X2) | two-factor enhancement |
| *q*(X1∩X2) = *q*(X1) + *q*(X2) | independent |
| *q*(X1∩X2) > *q*(X1) + q(X2) | nonlinear enhancement |

**3.4 Drivers mechanism of spatial-temporal variation of cropland abandonment**

This paper uses the regression analysis method to do linear regression analysis method of the influencing factors and dependent variables screened out by the above methods to explore the driving mechanism of spatiotemporal variation of CAR in the study area, and in the selection of regression model, choose to use stepwise regression method, this model can solve the problem of population、economics environment factors＇multi-factor multicollinearity. The linear regression equation is shown in equation (4). The regression coefficients were calculated based on the data of the independent variables and the dependent variable by the least square method. The regression coefficients indicate the importance of each index. The regression equation reflects the relationship between the independent variables and the dependent variable.

$y=a+b_{1}x_{1}+b_{2}x_{2}+\cdots+b_{k}x_{k}$ (4)

where $a$ is a constant, $b$ is the regression coefficient, $x$ is the impact factor, and $y$is the rate of cropland abandonment.

3. *Inadequate data presentation: The article mentions the selection of 19 key factors from population, economic environment, cropland attributes, and farming conditions, but it does not provide a clear explanation of the rationale behind the selection of these factors. Additionally, the article lacks specific data on the values and trends of the selected factors, making it difficult to assess their impact on cropland abandonment.*

**Reply**：

Thank you for reviewing the manuscript so carefully. These indicators are directly related factors selected based on the research results of relevant literature and the geographical environmental characteristics of this region. The explanations for the selected indicators are detailed in the 'description' column of Table 1. The indicators in the indicator system are used to identify the main driving factors of changes in the rate of arable land abandonment. The analysis of time-varying driving factors and spatially varying driving factors in the methodology is designed to illustrate the importance of these indicators.

**modified content:**

Combining the current research results and the geographical characteristics of the region, in order to better analyze the driving mechanism of cropland abandonment in KPEYC. The study based on the P-S-R（Pressure-State-Response）model, comprehensively considered the influencing factors from the different dimensions of pressure, state, and response, and refined the influencing factors, and constructed an indicator system with a total of 18 drivers selected from the four aspects of population, economic environment, cropland attributes, and farming conditions (Table 1).

**Table 1.** Drivers of cropland abandonment

| **Level**  **Indicators** | **Level 2**  **Indicators** | **Unit** | **Description** |
| --- | --- | --- | --- |
| **Population** | Year-end total population X1 | *Million people*  *Million people*  *Million people*  *Million people* | The larger the population, the less likely it is that cropland will be left fallow. **^[1]^** |
|  | Number of students in primary and secondary schools X2 |  | It can be used as supplementary labor for farming. **^[14]^** |
|  | Rural workers X3 |  | The lower the amount of labor, the higher the likelihood of abandonment. **^[5]^** |
|  | Agricultural population X4 |  | A decrease in the rural agricultural population makes abandonment more likely. **^[1]^** |
| **Economics**  **Environment** | Gross value of agricultural production X5 | *Billion yuan*  *Billion yuan*  *Billion yuan*  *Ten thousand yuan* | The greater the value of agricultural production, the more likely it is to be abandoned. **^[4]^** |
|  | Gross value of industrial production X6 |  | The more developed the industry is, the more likely it is to be abandoned. **^[7]^** |
|  | Gross tertiary industrial production X7 |  | The larger the output value of tertiary industry is, the more likely it is to be abandoned. **^[13]^**. |
|  | Per capita net income of farmers X8 |  | The higher the income of farmers, the higher the likelihood of abandonment. **^[5]^** |
| **Cropland**  **Attributes** | Soil thickness X9 | *Meter* | The thicker the soil layer, the less likely it is to be abandoned |
|  | Soil organic matter content X10 | *Grams per kilogram* | The higher the quality of the soil, the less likely it is to be abandoned. **^[5]^** |
|  | Average sunshine duration X11 | *Hour* | The better the light, the less likely it is to be abandoned. |
|  | Average air temperature X12 | *Celsius* | The better the temperature, the less likely to be abandoned. **^[9]^** |
|  | Average precipitation X13 | *Millimeter* | The richer the water resources, the less likely it is to be abandoned. **^[9]^** |
| **Farming**  **Conditions** | Elevation X14 | *Kilometer* | The higher the altitude, the more prone to abandonment. **^[14]^** |
|  | Slope X15 | *Degree* | The steeper the slope, the more likely it is to be abandoned. **^[11]^** |
|  | Slope direction X16 | *Degrees* | The sunnier the slope, the better the light, the less likely it is to be abandoned. **^[14]^** |
|  | Traffic mileage X17 | *Thousand Kilometers* | Poor access to cropland can lead to abandonment. **^[11]^** |
|  | Agriculture machinery powerX18 | *Million Watts* | The lower the degree of mechanization, the higher the likelihood of abandonment. **^[5]^** |

4. *Lack of discussion on limitations: The article does not acknowledge or discuss the limitations of the study. Every research project has inherent limitations, and it is important to address them to provide a balanced perspective. This omission reduces the credibility and reliability of the research findings.*

**Reply**：

Thanks for your valuable counsel. Based on your suggestions, we have revised the discussion section to analyze the advantages and limitations of the method used in this paper. The limitations discussed in this paper primarily focus on the constraints of the methodology and the generalizability of the results.

**modified content:**

The geodetector method leverages the similarity of two-dimensional spatial patterns to discern relationships among variables, which is a more stringent approach than merely assessing one-dimensional linear relationships. Additionally, grounded in the principles of spatial autocorrelation, this method enjoys the benefits of not requiring robust assumptions and being resilient against issues of covariance ^[25,26]^. Nonetheless, it remains challenging for the detector to fully eliminate the influence of confounding factors on variable relationships when used in isolation, representing a limitation in its application. Given that cropland abandonment is a more intricate issue than a singular social or natural geographic phenomenon, and building on existing research, this paper adopts a synergistic approach using Spearman correlation analysis, geodetector, and regression analysis techniques. This composite methodology allows for a more effective portrayal of the spatial and temporal dynamics of cropland abandonment within the KPEYC, as well as enabling a quantitative assessment of how driving factors impact ecological vulnerability.

This study still harbors some uncertainties. On one hand, the driving mechanism of cropland abandonment in the KPEYC region is intricate, and a consolidated indicator system for dissecting the forces behind this phenomenon has not been established yet. As a result, there are issues with multicollinearity among certain factors when constructing the indicator system, although this problem can be adequately resolved through methodological approaches. On the other hand, the applicability of the driving mechanism of cropland abandonment established in this paper for the KPEYC area to other ecologically fragile regions is considered. The KPEYC is situated in China's remote southwestern areas, where the agricultural environment is inferior to that of the ACA regions in the east and center, and the economic context is less advanced than that of the ACA in the southeast. Therefore, the issue of cropland abandonment in the KPEYC is more pronounced and poses greater management challenges compared to other regions.

5. *Limited generalizability: The study focuses specifically on the Karst Plateau in Eastern Yunnan, China, and the findings may not be applicable to other regions or contexts. The article does not discuss the potential implications or generalizability of the findings, limiting the broader relevance of the research.*

**Reply**：

Thank you for reviewing the manuscript so carefully. In response to your suggestion, we have added a second paragraph to the discussion about the generalizability of the results of this paper.

**added content**：

This study still harbors some uncertainties. On one hand, the driving mechanism of cropland abandonment in the KPEYC region is intricate, and a consolidated indicator system for dissecting the forces behind this phenomenon has not been established yet. As a result, there are issues with multicollinearity among certain factors when constructing the indicator system, although this problem can be adequately resolved through methodological approaches. On the other hand, the applicability of the driving mechanism of cropland abandonment established in this paper for the KPEYC area to other ecologically fragile regions is considered. The KPEYC is situated in China's remote southwestern areas, where the agricultural environment is inferior to that of the ACA regions in the east and center, and the economic context is less advanced than that of the ACA in the southeast. Therefore, the issue of cropland abandonment in the KPEYC is more pronounced and poses greater management challenges compared to other regions.

6. *Absence of policy recommendations: Despite mentioning that the study can provide decision support for preventing and controlling cropland abandonment, the article does not provide any specific policy recommendations or practical implications based on the research findings. This omission reduces the practical applicability and impact of the study*.

**Reply**：

Thank you for reviewing the manuscript so carefully. We have added the content about the policy recommendations on the Karst Plateau in Eastern Yunnan, China, in the discussion section.

**added content**：

Based on the research presented in this paper, we can gain an understanding of the current state of cropland abandonment in the KPEYC, which provides corresponding data support for future cropland protection policies and offers suggestions for reducing local cropland abandonment and safeguarding food production. Firstly, in response to the severe ecological and environmental issues in the area, it is imperative to solidly promote ecological restoration and management, optimize the ecological spatial pattern, and address serious karstification to achieve sustainable development in terms of ecological security. Secondly, facing the significant issue of "hollowing out" in rural areas, implementing rural revitalization strategies by integrating "agriculture + tourism" to develop the tertiary industry in rural areas is crucial. This involves promoting cropland transfers and consolidation according to local conditions, advancing local urbanization, improving policies that benefit farmers, and enhancing farming conditions to increase farmers' enthusiasm for production and promote the rational use of cropland. There is a need to establish a long-term mechanism to correct the problem of cropland abandonment; to orderly and lawfully encourage land transfers, guiding farmers who are unable to farm to transfer their land to operational entities, and to encourage regions with the right conditions to carry out market-based land transfer operations to revitalize cropland resources and prevent the waste of land assets. Thirdly, while governments at all levels have a preliminary understanding of the rectification of cropland abandonment and have achieved initial success, the protection of cropland resources is a long-term process aimed at promoting their rational use. To sustainably motivate farmers' eagerness to grow crops, it is necessary to establish a long-term early warning and remediation mechanism for cropland abandonment to prevent the resurgence of this phenomenon.

**Reviewer** # **3:**

1. *Justification of the statement: Page 4, paragraph 2. The given statement must have to justify with previous studies [1-3] as “The survey method is suitable for discovering the driving factors of geographical phenomena [1,2]”***.**

**Reply:**

Thanks for your questions. In the first part of the introduction, that is, citing references [1-3] is first a brief introduction to the situation of the abandonment of cropland in some areas of China, to study the most common reasons for the emergence of the abandonment of cropland in China. In the second paragraph, the current status of research on cropland abandonment is studied from both domestic and international perspectives, and the causes of cropland abandonment vary from region to region.

2. *However, the artilce does not detail the criteria for selecting these factors or their individual contributions, which would be crucial for assessing the study analytical depth.*

**Reply:**

Thanks for your questions. Taking into account the comments you have given, we have made changes in the construction of the indicator system.

**modified content:**

3.1 Selection of drivers

Combining the current research results and the geographical characteristics of the region, in order to better analyze the driving mechanism of cropland abandonment in KPEYC. The study based on the P-S-R（Pressure-State-Response）model, comprehensively considered the influencing factors from the different dimensions of pressure, state, and response, and refined the influencing factors, and constructed an indicator system with a total of 18 drivers selected from the four aspects of population, economic environment, cropland attributes, and farming conditions (Table 1).

**Table 1.** Drivers of cropland abandonment

| **Level**  **Indicators** | **Level 2**  **Indicators** | **Unit** | **Description** |
| --- | --- | --- | --- |
| **Population** | Year-end total population X1 | *Million people*  *Million people*  *Million people*  *Million people* | The larger the population, the less likely it is that cropland will be left fallow. **^[1]^** |
|  | Number of students in primary and secondary schools X2 |  | It can be used as supplementary labor for farming. **^[14]^** |
|  | Rural workers X3 |  | The lower the amount of labor, the higher the likelihood of abandonment. **^[5]^** |
|  | Agricultural population X4 |  | A decrease in the rural agricultural population makes abandonment more likely. **^[1]^** |
| **Economics**  **Environment** | Gross value of agricultural production X5 | *Billion yuan*  *Billion yuan*  *Billion yuan*  *Ten thousand yuan* | The greater the value of agricultural production, the more likely it is to be abandoned. **^[4]^** |
|  | Gross value of industrial production X6 |  | The more developed the industry is, the more likely it is to be abandoned. **^[7]^** |
|  | Gross tertiary industrial production X7 |  | The larger the output value of tertiary industry is, the more likely it is to be abandoned. **^[13]^**. |
|  | Per capita net income of farmers X8 |  | The higher the income of farmers, the higher the likelihood of abandonment. **^[5]^** |
| **Cropland**  **Attributes** | Soil thickness X9 | *Meter* | The thicker the soil layer, the less likely it is to be abandoned |
|  | Soil organic matter content X10 | *Grams per kilogram* | The higher the quality of the soil, the less likely it is to be abandoned. **^[5]^** |
|  | Average sunshine duration X11 | *Hour* | The better the light, the less likely it is to be abandoned. |
|  | Average air temperature X12 | *Celsius* | The better the temperature, the less likely to be abandoned. **^[9]^** |
|  | Average precipitation X13 | *Millimeter* | The richer the water resources, the less likely it is to be abandoned. **^[9]^** |
| **Farming**  **Conditions** | Elevation X14 | *Kilometer* | The higher the altitude, the more prone to abandonment. **^[14]^** |
|  | Slope X15 | *Degree* | The steeper the slope, the more likely it is to be abandoned. **^[11]^** |
|  | Slope direction X16 | *Degrees* | The sunnier the slope, the better the light, the less likely it is to be abandoned. **^[14]^** |
|  | Traffic mileage X17 | *Thousand Kilometers* | Poor access to cropland can lead to abandonment. **^[11]^** |
|  | Agriculture machinery powerX18 | *Million Watts* | The lower the degree of mechanization, the higher the likelihood of abandonment. **^[5]^** |

3. *the study highlights the core driving forces and contributing factors to CAR, it could benefit from a more nuanced discussion on how these factors interact and the potential for mitigating negative impacts through policy interventions.*

**Reply:**

Thanks for your questions. Section 3 of the Discussion section analyzes the interactions of these factors, and policy recommendations for mitigating cropland abandonment.

**added content:**

Based on the research presented in this paper, we can gain an understanding of the current state of cropland abandonment in the KPEYC, which provides corresponding data support for future cropland protection policies and offers suggestions for reducing local cropland abandonment and safeguarding food production. Firstly, in response to the severe ecological and environmental issues in the area, it is imperative to solidly promote ecological restoration and management, optimize the ecological spatial pattern, and address serious karstification to achieve sustainable development in terms of ecological security. Secondly, facing the significant issue of "hollowing out" in rural areas, implementing rural revitalization strategies by integrating "agriculture + tourism" to develop the tertiary industry in rural areas is crucial. This involves promoting cropland transfers and consolidation according to local conditions, advancing local urbanization, improving policies that benefit farmers, and enhancing farming conditions to increase farmers' enthusiasm for production and promote the rational use of cropland. There is a need to establish a long-term mechanism to correct the problem of cropland abandonment; to orderly and lawfully encourage land transfers, guiding farmers who are unable to farm to transfer their land to operational entities, and to encourage regions with the right conditions to carry out market-based land transfer operations to revitalize cropland resources and prevent the waste of land assets. Thirdly, while governments at all levels have a preliminary understanding of the rectification of cropland abandonment and have achieved initial success, the protection of cropland resources is a long-term process aimed at promoting their rational use. To sustainably motivate farmers' eagerness to grow crops, it is necessary to establish a long-term early warning and remediation mechanism for cropland abandonment to prevent the resurgence of this phenomenon.

4. *However, the article does not explicitly discuss the implications for local communities, food security, and ecological sustainability, which are critical for translating research findings into actionable strategies*.

**Reply:**

Thank you for reviewing the manuscript so carefully. We have added the content about the policy recommendations on the Karst Plateau in Eastern Yunnan, China, in the discussion section.

**added content**：

According to the results of this study, it can still help the relevant departments to understand the status quo of regional cropland abandonment and provide corresponding data support for future cropland protection policies. It is necessary to create a different kind of countryside for “agriculture+ tourism”, promote land transfer and concentration according to local conditions, local urbanization, improve agricultural policies, and improve farming conditions in order to increase farmers' enthusiasm for production and promote the rational use of cropland; it is necessary to solidly push forward ecological restoration and governance, optimize the ecological spatial pattern, manage the serious areas of rocky desertification, and appropriately alleviate the contradiction between people and land. To establish a long-term mechanism to rectify the abandonment of cropland; to orderly promote the transfer of land in accordance with the law, to guide farmers who are not in a position to carry out normal farming to transfer land to the main body of the operation, and to encourage conditional places to carry out the market operation of land transfer, to revitalize the cropland resources, and to avoid the waste of land resources; at present, the governments of various levels of the abandonment of cropland have had a preliminary understanding of the rectification of cropland abandonment has had initial results, but the protection of cropland resources is a long-term process, so as to promote the rational use of cropland. The protection of cropland resources is a long-term process, to fully mobilize the enthusiasm of farmers to grow food, it is necessary to establish a long-term early warning of cropland abandonment, remediation mechanism, to put an end to the phenomenon of cropland abandonment of the resurgence of the phenomenon.

5. *Limitations and Future Research: While the article indicates a comprehensive analysis, it does not address the limitations of the study, such as potential biases in data selection, the resolution of spatial data, or the generalizability of the findings to other karst regions. Future research directions could include exploring the socio-economic impacts of cropland abandonment on local communities, integrating ecological considerations into economic analyses, and developing adaptive management strategies that address both economic and environmental sustainability.*

**Reply:**

Thanks for your questions. In light of your recommendations, the discussion section has been revised to address individual aspects: the limitations of the methodology, the generalizability of the results, and policy recommendations. The first part primarily introduces the strengths and limitations of the methods used in this paper for analyzing cropland abandonment; the second point discusses the issues with constructing indicators and the generalizability of the research findings; the third part presents policy recommendations based on the analysis results.

**added content:**

The geodetector method leverages the similarity of two-dimensional spatial patterns to discern relationships among variables, which is a more stringent approach than merely assessing one-dimensional linear relationships. Additionally, grounded in the principles of spatial autocorrelation, this method enjoys the benefits of not requiring robust assumptions and being resilient against issues of covariance ^[25,26]^. Nonetheless, it remains challenging for the detector to fully eliminate the influence of confounding factors on variable relationships when used in isolation, representing a limitation in its application. Given that cropland abandonment is a more intricate issue than a singular social or natural geographic phenomenon, and building on existing research, this paper adopts a synergistic approach using Spearman correlation analysis, geodetector, and regression analysis techniques. This composite methodology allows for a more effective portrayal of the spatial and temporal dynamics of cropland abandonment within the KPEYC, as well as enabling a quantitative assessment of how driving factors impact ecological vulnerability.

This study still harbors some uncertainties. On one hand, the driving mechanism of cropland abandonment in the KPEYC region is intricate, and a consolidated indicator system for dissecting the forces behind this phenomenon has not been established yet. As a result, there are issues with multicollinearity among certain factors when constructing the indicator system, although this problem can be adequately resolved through methodological approaches. On the other hand, the applicability of the driving mechanism of cropland abandonment established in this paper for the KPEYC area to other ecologically fragile regions is considered. The KPEYC is situated in China's remote southwestern areas, where the agricultural environment is inferior to that of the ACA regions in the east and center, and the economic context is less advanced than that of the ACA in the southeast. Therefore, the issue of cropland abandonment in the KPEYC is more pronounced and poses greater management challenges compared to other regions.

Based on the research presented in this paper, we can gain an understanding of the current state of cropland abandonment in the KPEYC, which provides corresponding data support for future cropland protection policies and offers suggestions for reducing local cropland abandonment and safeguarding food production. Firstly, in response to the severe ecological and environmental issues in the area, it is imperative to solidly promote ecological restoration and management, optimize the ecological spatial pattern, and address serious karstification to achieve sustainable development in terms of ecological security. Secondly, facing the significant issue of "hollowing out" in rural areas, implementing rural revitalization strategies by integrating "agriculture + tourism" to develop the tertiary industry in rural areas is crucial. This involves promoting cropland transfers and consolidation according to local conditions, advancing local urbanization, improving policies that benefit farmers, and enhancing farming conditions to increase farmers' enthusiasm for production and promote the rational use of cropland. There is a need to establish a long-term mechanism to correct the problem of cropland abandonment; to orderly and lawfully encourage land transfers, guiding farmers who are unable to farm to transfer their land to operational entities, and to encourage regions with the right conditions to carry out market-based land transfer operations to revitalize cropland resources and prevent the waste of land assets. Thirdly, while governments at all levels have a preliminary understanding of the rectification of cropland abandonment and have achieved initial success, the protection of cropland resources is a long-term process aimed at promoting their rational use. To sustainably motivate farmers' eagerness to grow crops, it is necessary to establish a long-term early warning and remediation mechanism for cropland abandonment to prevent the resurgence of this phenomenon.

**Reviewer #4:**

1. *The discussion part should be polished by comparing the findings with the existing studies.*

**Reply:**

Thanks for your questions. In light of your recommendations, the discussion section has been revised to address individual aspects: the limitations of the methodology, the generalizability of the results, and policy recommendations. The first part primarily introduces the strengths and limitations of the methods used in this paper for analyzing cropland abandonment; the second point discusses the issues with constructing indicators and the generalizability of the research findings; the third part presents policy recommendations based on the analysis results.

**added content:**

The geodetector method leverages the similarity of two-dimensional spatial patterns to discern relationships among variables, which is a more stringent approach than merely assessing one-dimensional linear relationships. Additionally, grounded in the principles of spatial autocorrelation, this method enjoys the benefits of not requiring robust assumptions and being resilient against issues of covariance ^[25,26]^. Nonetheless, it remains challenging for the detector to fully eliminate the influence of confounding factors on variable relationships when used in isolation, representing a limitation in its application. Given that cropland abandonment is a more intricate issue than a singular social or natural geographic phenomenon, and building on existing research, this paper adopts a synergistic approach using Spearman correlation analysis, geodetector, and regression analysis techniques. This composite methodology allows for a more effective portrayal of the spatial and temporal dynamics of cropland abandonment within the KPEYC, as well as enabling a quantitative assessment of how driving factors impact ecological vulnerability.

This study still harbors some uncertainties. On one hand, the driving mechanism of cropland abandonment in the KPEYC region is intricate, and a consolidated indicator system for dissecting the forces behind this phenomenon has not been established yet. As a result, there are issues with multicollinearity among certain factors when constructing the indicator system, although this problem can be adequately resolved through methodological approaches. On the other hand, the applicability of the driving mechanism of cropland abandonment established in this paper for the KPEYC area to other ecologically fragile regions is considered. The KPEYC is situated in China's remote southwestern areas, where the agricultural environment is inferior to that of the ACA regions in the east and center, and the economic context is less advanced than that of the ACA in the southeast. Therefore, the issue of cropland abandonment in the KPEYC is more pronounced and poses greater management challenges compared to other regions.

Based on the research presented in this paper, we can gain an understanding of the current state of cropland abandonment in the KPEYC, which provides corresponding data support for future cropland protection policies and offers suggestions for reducing local cropland abandonment and safeguarding food production. Firstly, in response to the severe ecological and environmental issues in the area, it is imperative to solidly promote ecological restoration and management, optimize the ecological spatial pattern, and address serious karstification to achieve sustainable development in terms of ecological security. Secondly, facing the significant issue of "hollowing out" in rural areas, implementing rural revitalization strategies by integrating "agriculture + tourism" to develop the tertiary industry in rural areas is crucial. This involves promoting cropland transfers and consolidation according to local conditions, advancing local urbanization, improving policies that benefit farmers, and enhancing farming conditions to increase farmers' enthusiasm for production and promote the rational use of cropland. There is a need to establish a long-term mechanism to correct the problem of cropland abandonment; to orderly and lawfully encourage land transfers, guiding farmers who are unable to farm to transfer their land to operational entities, and to encourage regions with the right conditions to carry out market-based land transfer operations to revitalize cropland resources and prevent the waste of land assets. Thirdly, while governments at all levels have a preliminary understanding of the rectification of cropland abandonment and have achieved initial success, the protection of cropland resources is a long-term process aimed at promoting their rational use. To sustainably motivate farmers' eagerness to grow crops, it is necessary to establish a long-term early warning and remediation mechanism for cropland abandonment to prevent the resurgence of this phenomenon.

2. *The conclusion should be shorten and be very specific.*

**Reply:**

Thanks for your questions. The conclusion section has been modified.

**modified content:**

6 Conclusion

To analyze the spatial-temporal patterns in KPEYC, this study used correlation analysis and geodetector to identify the main factors that have driven the cropland abandonment in this region over time and space. By applying linear regression analysis to explore the driving mechanisms behind the spatial-temporal changes in cropland abandonment. The following conclusions were reached:

(1) The CAR in KPEYC shows a fluctuating trend, with an average value of 9.78%, and a spatial distribution pattern of "high in the center and low in the north and south". From 2010 to 2015, the most prominent phenomenon of cropland abandonment occurred, and the situation improved in Wenshan Prefecture after 2015. Compared to the other two areas, Zhaotong City has a lower CAR.

(2) In terms of temporal changes, the economic environment and farming conditions have obvious correlations with cropland abandonment. GDP, gross value of agricultural production, and gross value of industrial production in the economic environment have the largest correlation coefficients with CAR, and mechanical power has the highest correlation with changes in abandonment rates in farming conditions.

(3) Gross tertiary industrial production, gross value of industrial production, and GDP have the strongest explanatory power for the spatial distribution of cropland abandonment, followed by soil thickness.

(4) GDP, gross value of agricultural production, and gross tertiary industrial production are the main driving factors of spatial and temporal changes of CAR: the larger the GDP, the higher the CAR; the higher the gross value of agricultural production and gross tertiary industrial production, the lower the CAR. Elevation, soil thickness, and traffic mileage are the main driving factors of the spatial and temporal changes of the CAR.

3. *The figures came with low resolution and not understandable. Please upload high-resolution figures with the revised version of the manuscript.*

**Reply:**

Thanks for your questions. We have uploaded high-resolution figures with the revised version of the manuscript. The resolution of the modified is 300 dpi.

**modified content:**

**Figures:**

**Fig. 1.** The location of the study area. (a) The topographic map. (b) The KPEYC in China. (c) The 20-year change of total grain output and GDP.

**Fig.** **2.** Distribution of abandoned cropland area and cropland abandonment rate. (a) The changes of the abandoned cropland area in each city. (b) The changes in the cropland abandoned area and the cropland abandonment rate in KPEYC in the past 20 years.

**Fig.3.** Distribution of cropland abandonment rate in KPEYC during 2001-2020.

**Fig.4.** The correlation between the change factors and the cropland abandonment rate in KPEYC.

**Fig. 5.** Drivers interactive detection results of the mean value in the past 20 years. (a) The interactive detection results of factors. (b) High-value graph of interactive detection.
