# Supplementary material for: Spatial-temporal characterization of cropland abandonment and its driving mechanisms in the Karst Plateau in Eastern Yunnan, China, 2001–2020
Source: PLoS One. 2024 Jul 17;19(7):e0307148. doi: 10.1371/journal.pone.0307148 (PMC11253946; doi:10.1371/journal.pone.0307148)
Supplement: Attachment — Submitted filename: Response to Reviewers.docx [file pone.0307148.s002.docx]

Dear Editor:

We really appreciate all of the insightful and constructive comments from the reviewers. We have thoroughly revised the manuscript (PONE-D-23-43715R1) according to all comments received. In our point-by-point response attached below, reviewers’ comments is in larger italic fonts (12 pt.) and our response is in smaller fonts (10 pt.). We hope that the revised version would be closer for publication in your journal.

Look forward to hearing from you. Thank you.

With best wishes,

Yours sincerely,

Jiasheng Wang

Response to reviewers’ comments on our manuscript

PONE-D-23-43715R1

**Reviewer #1**

1. *Variable Selection Rationale: Please elaborate on how the selected variables directly contribute to addressing the study’s objectives and hypotheses. This would strengthen the logical flow and foundational basis of your analysis.*

**Reply:**

We've added information about the variable selection rationale in line 186. The content outlines the process of selecting impact factors for the study of cropland abandonment.

**Added content**:

Cropland abandonment results from human utilization of cropland, which in turn is influenced by human decision-making. Making informed decisions requires a comprehensive consideration of both human and land factors. Human factors such as labor force and economic income play a crucial role in determining whether or not to abandon cropland, while land factors such as the attributes of farmland and farming conditions influence the location of abandonment. Given that this paper's research unit is the county, we have selected population, economic environment, cropland attributes, and farming conditions as the key influencing factors to be analyzed.

2. *Limitations Discussion: Expand the discussion of the study's limitations, addressing potential biases and data resolution issues explicitly. This will add to the credibility and scholarly integrity of your findings.*

**Reply:**

Thanks for your suggestion. We added a limitations discussion in line 484. This section discusses the study's methodology, data, and research scale limitations. The content is as follows.

**Added content**:

This research analyzes the influencing factors and possible mechanisms of cropland abandonment using correlation coefficients and geodetector, and achieves certain results. However, there are some limitations that may introduce uncertainties into the results. First of all, the data used in this study were obtained from statistical yearbooks and remote sensing products. These data may have some errors, which could lead to inaccuracies in the results. When extracting abandoned cropland, there may be a mixed pixel that is still recognized as abandoned plots, resulting in an overestimation of the actual area of abandoned cropland. This could have a certain impact on the results of the study. Second, the analysis is limited to the county level, and all indicators are the result of county-wide statistics, which could ignore internal differences. In the future, it would be interesting to explore the impact factors of cropland abandonment at smaller spatial scales. Finally, the results of the geodetector method are influenced by the way spatial partitioning is done, but there is currently no widely recognized and reliable method for spatial partitioning. It would be necessary to conduct quantitative research on the effects of spatial partitioning on geodetector in the future.

3. *Data Presentation: Ensure all figures are of high resolution and data presentations are accessible, aiding in the clear communication of your results and analyses.*

**Reply:**

Thank you for your review and comments. We have uploaded high-resolution figures with the revised version of the manuscript. The resolution of the modified is 600 dpi.

**modified content:**

Fig. 1. The location of the study area. (a) The topographic map. (b) The KPEYC in China. (c) The 20-year change of total grain output and GDP.

Fig. 2. Distribution of abandoned cropland area and cropland abandonment rate. (a) The changes of the abandoned cropland area in each city. (b) The changes in the cropland abandoned area and the cropland abandonment rate in KPEYC in the past 20 years.

Fig.3. Distribution of cropland abandonment rate in KPEYC during 2001-2020.

Fig.4. The correlation between the change factors and the cropland abandonment rate in KPEYC.

Fig. 5. Drivers interactive detection results of the mean value in the past 20 years. (a) The interactive detection results of factors. (b) High-value graph of interactive detection.

4. *Implications and Generalizability: Discuss the broader implications of your findings, particularly how they might apply to other regions or contexts, to enhance the manuscript's relevance and impact.*

**Reply:**

Thanks for your suggestion. We added implications and generalizability in line 469. The results of the study are first described, the findings of this piece are compared with those of other regions, and their generalisability is discussed. The content is as follows.

**modified content:**

The research conducted on the issue of CAR in KPEYC reveals that economic factors play a pivotal role in influencing this phenomenon. These economic factors directly impact the reduction in agricultural income and the increase in farmers' consumption levels, thereby affecting their decision to abandon their lands. This finding resonates with the principles outlined in von Thünen's rent theory[25,26], which posits that the marginalization of land due to its poor suitability and diminishing economic returns underlie the decision to abandon land. However, it diverges from the findings of Han Ze[27], who highlighted that cropland abandonment in the karst mountains of Guizhou-Guangxi, China, is predominantly driven by agricultural practices and soil conditions, with soil erosion being a critical determinant of its distribution. The discrepancy could be attributed to the regional variations in the spatial distribution of cropland abandonment or possibly influenced by the different stages of regional economic development; for instance, Yunnan Province has exhibited higher GDP outputs compared to Guizhou Province over the past years.

The methodology employed in this research can indeed be applied across diverse regions globally; however, it is essential to recognize that varying data sets or distinct political cultures may yield different outcomes. For instance, when applied to countries outside of China, discrepancies in land management systems could potentially result in varying primary factors identified through the analysis.

5. *Policy Recommendations: Provide detailed policy recommendations based on your findings to demonstrate the practical applications of your research in addressing cropland abandonment.*

**Reply:**

Thanks for your suggestion. We added policy recommendations in line 458. Discussion in terms of economics, rocky desertification management and government policies. The content is as follows.

**modified content:**

Based on the findings of this study and in alignment with the management policies of the Yunnan Province area in China, the following recommendations are proposed to effectively tackle the issue of cropland abandonment and ensure food security in the region. To address the labor outflow issue induced by economic development, the following measures are suggested that: (i) Rural agricultural subsidies should be enhanced: This would provide much-needed financial support to farmers, especially those cultivating land in mountainous regions. (ii) Preferential treatments should be extended to farmers in marginal lands: For instance, by offering educational and medical benefits to the children of these farmers, thus alleviating the economic burden on their families. (iii) Financial risk mitigation for farmers: By providing low-interest loans and agricultural insurance, farmers in marginal mountainous areas can better withstand financial risks associated with agricultural production. (iv) Expanding sales channels through internet platforms: Utilizing online platforms to open up new sales channels and expand markets for agricultural produce, especially those that are less amenable to long-distance transportation, thereby reducing the sales risk for farmers. (v) Enhanced agricultural technology support: By improving seeds, providing advanced agricultural cultivation technologies, and boosting crop productivity.

For the severely rocky desertified areas in KPEYC, it is imperative to thoroughly implement the concept of an ecological civilization. This includes executing afforestation projects on mountaintops, developing specialized economic industries on the slopes, and undertaking the "slope-to-staircase" project to fully utilize arable land and enhance land productivity. Such measures not only conserve water resources but also reduce soil erosion and tackle pest issues.

While governments at all levels have made preliminary progress in addressing the issue of abandoned cropland, the protection of cropland resources remains a long-term process aimed at promoting the rational use of these resources. To continuously stimulate farmers' enthusiasm for farming, it is necessary to establish a long-term early warning and remediation mechanism for abandoned cropland to prevent the recurrence of this phenomenon.
